# Puberty is a critical window for the impact of diet on mammary gland development in the rabbit

**DOI:** 10.1002/dvdy.91

**Published:** 2019-08-02

**Authors:** Cathy Hue‐Beauvais, Johann Laubier, Nicolas Brun, Inès Houtia, Florence Jaffrezic, Claudia Bevilacqua, Fabienne Le Provost, Madia Charlier

**Affiliations:** ^1^ GABI, INRA, AgroParisTech Université Paris‐Saclay Jouy‐en‐Josas France

**Keywords:** diet, mammary epithelial cells, mammary gland, puberty, rabbit

## Abstract

**Background:**

Nutritional changes can affect future lactation efficiency. In a rabbit model, an obesogenic diet initiated before puberty and pursued throughout pregnancy enhances mammary differentiation, but when started during the neonatal period can cause abnormal mammary development in early pregnancy. The aim of this study was to investigate the impact of an unbalanced diet administered during the pubertal period only.

**Results:**

Consuming an obesogenic diet at puberty did not affect either metabolic parameters or certain maternal reproductive parameters at the onset of adulthood. In contrast, at Day 8 of pregnancy, epithelial tissue showed a lower proliferation rate in obesogenic‐diet fed rabbits than in control‐diet fed rabbits. *Wap* and *Cx26* genes, mammary epithelial cell differentiation markers, were upregulated although Wap protein level remained unchanged. However, the expression of genes involved in lipid metabolism and in alveolar formation was not modified.

**Conclusion:**

Taken together, our results demonstrate that the consumption for 5 weeks of an obesogenic diet during the pubertal period initiates mammary structure modifications and affects mammary epithelial cell proliferation and differentiation. Our findings highlight the potentially important role played by unbalanced nutrition during critical early‐life windows in terms of regulating mammary epithelial cell differentiation and subsequent function in adulthood.

## Key Findings

- Our results demonstrate that the consumption for five weeks of an obesogenic diet during the pubertal period initiates mammary structure modifications and affects mammary epithelial cell proliferation and differentiation. Our findings highlight the potentially important role played by unbalanced nutrition during critical early‐life windows in terms of regulating mammary epithelial cell differentiation and subsequent function in adulthood.

## 1 INTRODUCTION

The mammary gland is a complex secretory organ that contains different cell types,^1^ one of the most important being mammary epithelial cells (MECs) which are responsible for the synthesis and secretion of milk components.^2^ The growth and differentiation of MEC are lengthy processes during the animal's lifespan (fetal, pre‐, and postpubertal periods) and reproductive cycles (pregnancy and lactation) and they occur under stage‐dependent hormonal and environmental control.^3^, ^4^ It has already been shown that diet can influence mammary gland development and subsequent milk production.^5^ Moreover, data using rodent models have revealed that nutrition during early life, when the mammary tissue is undergoing proliferation and extensive modeling, can alter susceptibility to mammary tumor development.^6^, ^7^ We previously showed in a rabbit model that an obesogenic diet (OD) initiated before puberty and pursued throughout puberty altered mammary gland development.^8^ The same diet also markedly modified milk composition and induced long‐term effects on mammary development and function.^9^, ^10^, ^11^

Few studies have highlighted the presence and the importance of critical periods during which nutrition can affect mammary development and function. One of the most widely studied periods is in utero, because maternal nutrition during pregnancy can influence mammary outcomes in both dams and offspring. Indeed, the consumption of a low protein diet during pregnancy was shown to decrease MEC development in rodents.^12^ Kucia et al showed that a high‐protein diet ingested by pregnant dams led to a reduction in mammary gland functional tissue as well as changes to the expression of genes involved in milk synthesis.^13^ Maternal diet‐induced obesity during both pregnancy and lactation negatively affected mammary gland function.^14^ Moreover, results showed that maternal exposure to protein restriction could change fatty acid metabolism in the mammary gland and thus modify milk composition.^15^ For these reasons, the neonatal period is also considered as critical through the consumption of maternal milk, the composition of which may vary depending on the dam's diet and thus have long‐term effects on offspring health. In a rabbit model, the ingestion of milk produced by dams fed an OD led to an increased adiposity in adulthood, as well as to impaired mammary function.^9^, ^10^

Puberty is also a period of upmost importance for mammary gland development, since the mammary gland develops from a rudimentary tree to a branched epithelial network of ducts. This process involves growth, proliferation, migration, branching, invasion, apoptosis, and most importantly, tight regulation which allows these processes to take place simultaneously. Such regulations depend on species and might thus result to different kinetics in mammary gland development^16^ and/or specific anatomical features, such as terminal end buds in rodents or terminal ductal‐lobular units in ruminants.^17^

When considering the period around puberty as a window of nutritional sensitivity, few studies have investigated its effects on mammary development; most have focused on tumorigenesis.^18^, ^19^, ^20^, ^21^ In a nontumor context, studies showed that the number, proliferation and differentiation of MEC could be altered by the diet consumed during puberty.^20^ Furthermore, the exposure of mice to a high‐fat diet during puberty caused profound and strain‐specific effects on body weight, adiposity, mammary gland development, and hormone responsiveness, thus highlighting the crucial importance of this period.^22^ Additional studies in mice indicated that short exposure during the peri‐pubertal period to a high‐fat diet irreversibly increased the development of mammary hyperplasia and mammary cell proliferation, and decreased tumor latency.^21^, ^23^ In cattle, overfeeding during puberty exerts a negative effect on growth of the mammary parenchyma and subsequent milk potential.^24^, ^25^, ^26^, ^27^ There is therefore considerable interest in understanding the importance of the pubertal period, because the mammary epithelium is rapidly invading the fat pad; it thus constitutes the starting point for later normal mammary functions in adulthood.^5^

The objective of the present study was therefore to determine the influence of an unbalanced diet, during the peri‐pubertal period exclusively, on mammary development and function in early pregnancy. We used a previously developed model of rabbits fed a high‐fat/high‐sugar diet that had displayed alterations to mammary development.^8^, ^9^ We analyzed the long‐term impacts of this OD consumed during puberty only, on body weight and metabolic profile in adulthood. We also examined maternal reproductive parameters during early pregnancy and described the mammary phenotype.

## 2 RESULTS

### 2.1 Physiological parameters of rabbits fed an OD

In order to highlight the importance of nutrition during the period surrounding puberty, we analyzed certain physiological parameters in two groups of rabbits fed with a control diet (CD) or OD during the 5 weeks challenge period between 8 and 13 weeks of age. We measured body weight and food intake over three periods: between weaning and the start of the nutritional challenge period (from 4 to 8 weeks of age) in order to observe the growth of rabbits under CD; during the nutritionally challenge period (from 8 to 13 weeks of age) in order to study the impact of both CD and OD throughout the peri‐pubertal period, and finally during adulthood (from 13 to 20.5 weeks of age, before mating) in order to observe the effect of the challenge period over the longer term.

**Figure 1:**
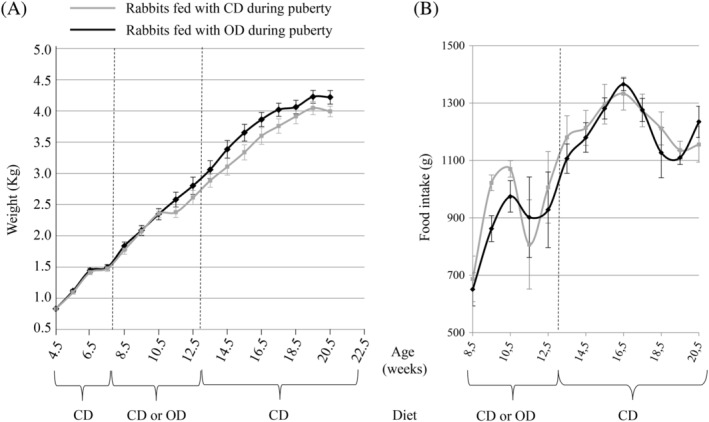
Average weight (A) and food intake (B) of female receiving either CD (n = 9) or OD (n = 7) during the peri‐pubertal period. Data are expressed as means ± SEM

When all the female rabbits were fed with CD from weaning to 7 weeks of age, no differences were observed in terms of weight gain (Figure 1A). Similarly, during puberty, no significant differences were measured between the two groups of rabbits. After the end of the nutritionally challenge period, all the females fed with CD, exhibited no difference in body weight gain. Moreover, the food intake did not differ between CD and OD groups (Figure 1B).

Metabolic profiles were analyzed in peripheral blood collected at 20 weeks of age, before mating (Table 1). No significant differences were observed between the two groups with respect to plasma cholesterol, triglyceride, glucose, and leptin levels.

**Table 1:** Metabolic profile of CD or OD‐fed dams at 20 weeks of age

	Cholesterol (mmol/L)	Triglycerides (mmol/L)	Glucose (mmol/L)	Leptin (ng/mL)
CD	0.45 ± 0.05	1.85 ± 0.80	1.96 ± 0.07	0.45 ± 0.12
OD	0.42 ± 0.04	1.61 ± 0.58	1.88 ± 0.11	0.53 ± 0.21
*P*‐value	NS^a^	NS	NS	NS

a NS, nonsignificant.

Taken together, these results showed that an OD administered during the pubertal period did not markedly affect either the weight curve or food intake. Moreover, this unbalanced diet did not affect the animal's metabolism at the onset of adulthood.

Similarly, maternal reproductive parameters such as pregnancy rate, litter size, and fetal mortality were not impacted by OD consumption during the pubertal period in our model (data not shown).

### 2.2 Effects of OD during puberty on the mammary gland in early pregnancy

#### 2.2.1 *Mammary gland morphology*

**Figure 2:**
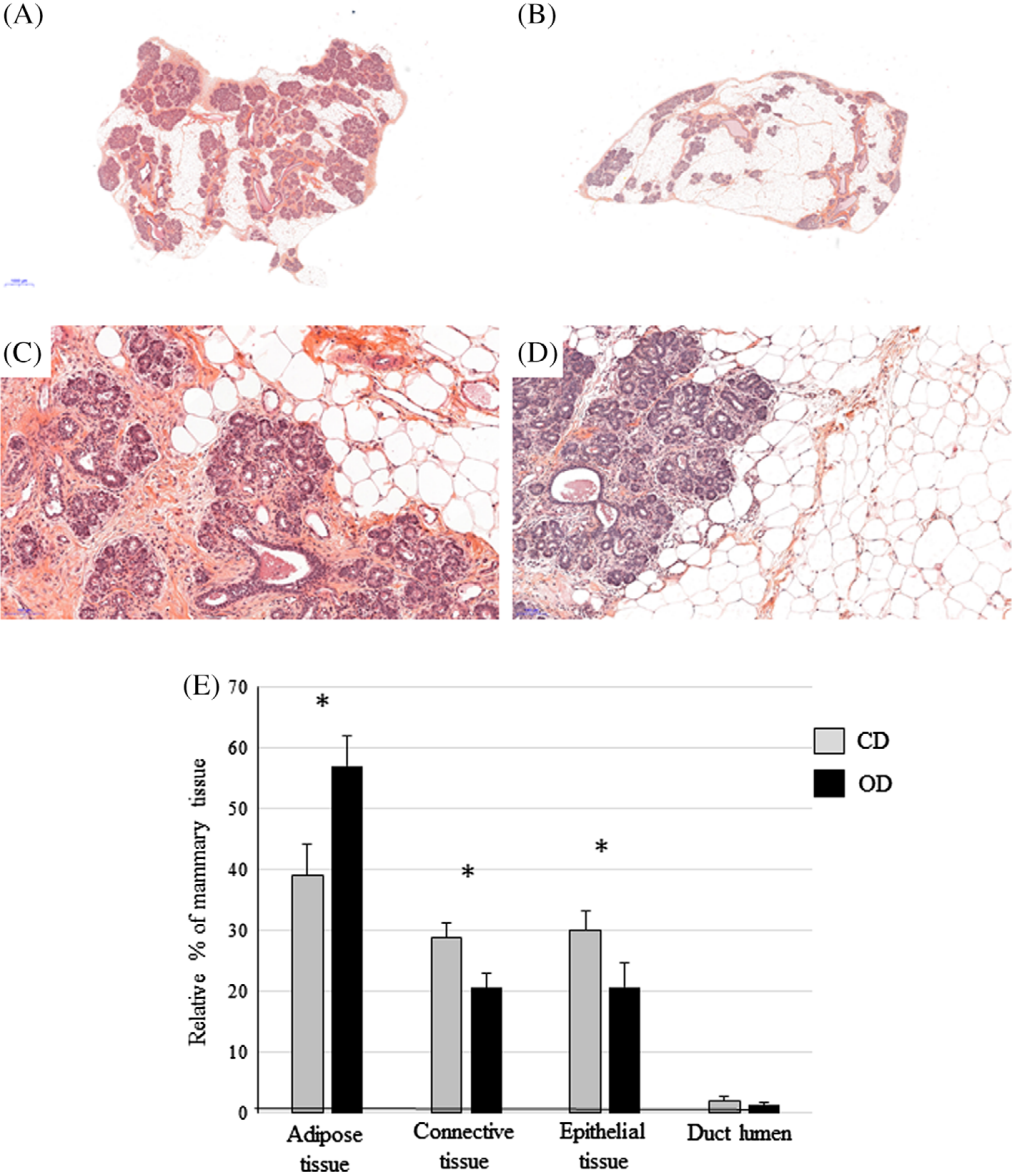
Histological analyses of mammary gland on Day 8 of pregnancy. Hematoxylin and eosin staining of 5‐μm mammary sections observed at different magnifications from CD (A,C) and OD (B,D) fed rabbits. Representative scans of total (A,B) and low magnified (C,D) sections. The scale bars represent 2 mm (A,B) and 100 μm (C,D). Relative quantification of mammary gland tissues (E). Data are expressed as means ± SEM. Significant differences (*P* < .05) between the groups are indicated by asterisks (*). Number of animals per group: CD: n = 9; OD: n = 7

To examine the effects of diet during puberty on the mammary gland, histological analyses were performed. The examination of mammary tissue sections from CD‐fed and OD‐fed rabbits on Day 8 of pregnancy revealed important differences between the two groups (Figure 2). The surface occupied by adipose tissue was higher in OD mammary glands, while connective and epithelial tissue surfaces were more abundant in the CD mammary glands. To further investigate these morphological differences, the relative areas of each tissue were evaluated using a quantitative analysis based on the ratio between epithelial, adipose, connective or lumen ducts and total section areas (Figure 2E). The ratio between duct lumen area and epithelial tissue has been compared between CD and OD mammary gland, and did not reflect changes in morphological assessment of the epithelial tissue. Mammary glands from the OD‐fed group displayed a larger relative area of adipose tissue compared to the CD group (57.2 ± 5.0 vs 39.0 ± 5.1% respectively*, P* = .0086). Conversely, the relative areas of epithelial and connective tissues were higher in CD mammary glands (connective tissue: 20.8 ± 2.9% vs 30.1 ± 3.1%, *P* = .04 and epithelial tissue: 20.7 ± 2.4% vs 28.8 ± 2.5%, *P* = .015, respectively; Figure 2E). However, regarding the lumens of mammary ducts (Figure 2C,D), no differences in the relative areas were observed between the CD and OD groups.

These findings support the observation that OD restricted to the period around puberty had markedly modified the mammary gland structure, and in particular, the relative proportions of epithelial and adipose tissues at early pregnancy.

#### 2.2.2 *MEC differentiation*

**Figure 3:**
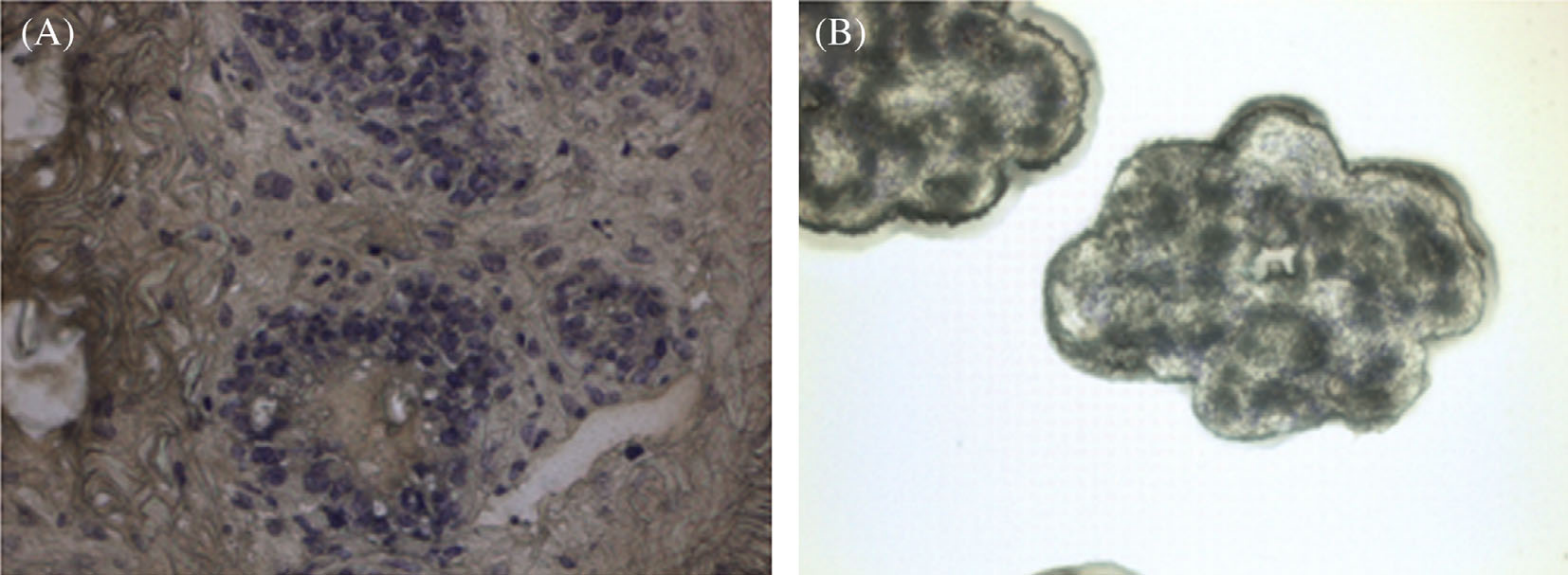
Mammary epithelial enrichment by laser capture microdissection. A, Cresyl violet‐stained rabbit mammary gland sections on Day 8 of pregnancy at low magnification (×20). The nuclei of epithelial and myoepithelial cells are in blue. B, High magnification (×120) of epithelial alveoli isolated by LCM

In order to analyze the impact of diet during the peri‐pubertal period on mammary epithelial tissue, we assessed specific MEC gene expression patterns using laser capture microdissection (LCM). This approach was chosen in order to isolate and characterize the MEC population as the efficiency of this technique has already been proven in numerous tissues, species or during cancer studies.^28^, ^29^, ^30^ Stained mammary sections (Figure 3A) capturing MEC (Figure 3B) provided highly enriched material for mRNA studies.

**Figure 4:**
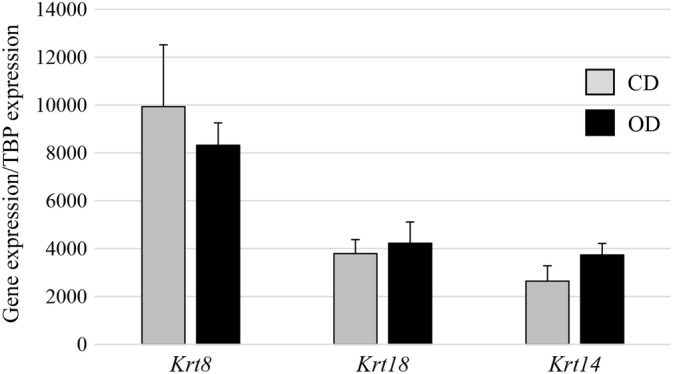
Characterization of mammary cells captured by LCM. Analyses were performed in the two groups of rabbits according to the diet received during the 8 to 13 week period (CD: n = 9; OD: n = 7). RT‐PCR were assessed for specific markers from myoepithelial cells (*Krt14*) and epithelial cells (*Krt8*). Data are expressed as means ± SEM

Because of the very close proximity of MEC and myoepithelial cells in the mammary gland, it is difficult to obtain a cell population exclusively made up of MEC. The mammary gland epithelium contains an inner layer of MEC and an outer layer of myoepithelial cells separating the epithelial layer from the extracellular matrix. Myoepithelial cells are involved in milk ejection during lactation and resemble smooth muscle cells containing specific cytokeratin filaments. To estimate the purity of the samples and consequently their putative contamination by myoepithelial cells, mRNA levels of cell‐specific markers were determined using RT‐qPCR. In order to characterize the MEC, *keratin 8* (*Krt8*) gene expression was measured and as for the myoepithelial cells, the *keratin 14* (*Krt14)* gene was used (Figure 4). We were successful in selectively capturing epithelial, cells as shown by the expression of *Krt8*, which is characteristic of these cells, and myoepithelial cells revealed by *Krt14* expression. This expression, which is similar in both groups suggest that the proportions of the various cell types in mammary epithelial tissue did not differ between the CD and OD‐fed dams (Figure 4).

**Figure 5:**
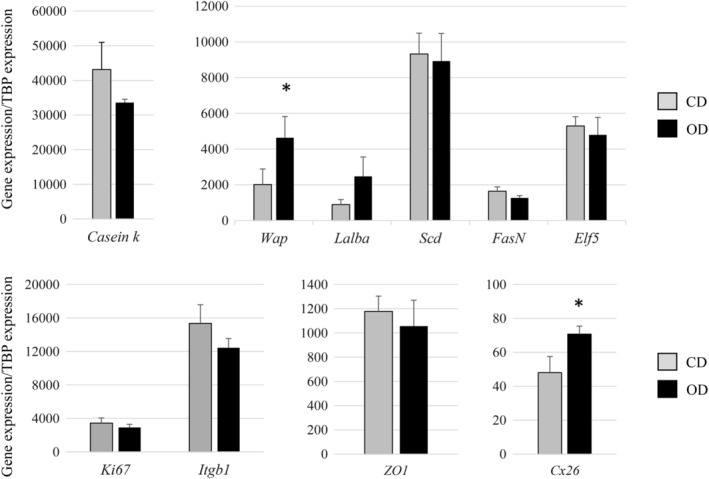
Expression of genes in MEC captured by LCM. Analyses were performed in the two groups of rabbits according to the diet received during the 8 to 13 week period (CD or OD). The expression of transcripts was assessed for *κ casein, Wap*, *Lalba*, *Scd*, *FasN*, *Elf5*, *Ki67*, *Itgb1*, *ZO‐1*, and *Cx26* and normalized with *Tbp* as the housekeeping gene. Data are expressed as means ± SEM. Significant differences (at least *P* < .05) between the groups are indicated by asterisk (*)

**Figure 6:**
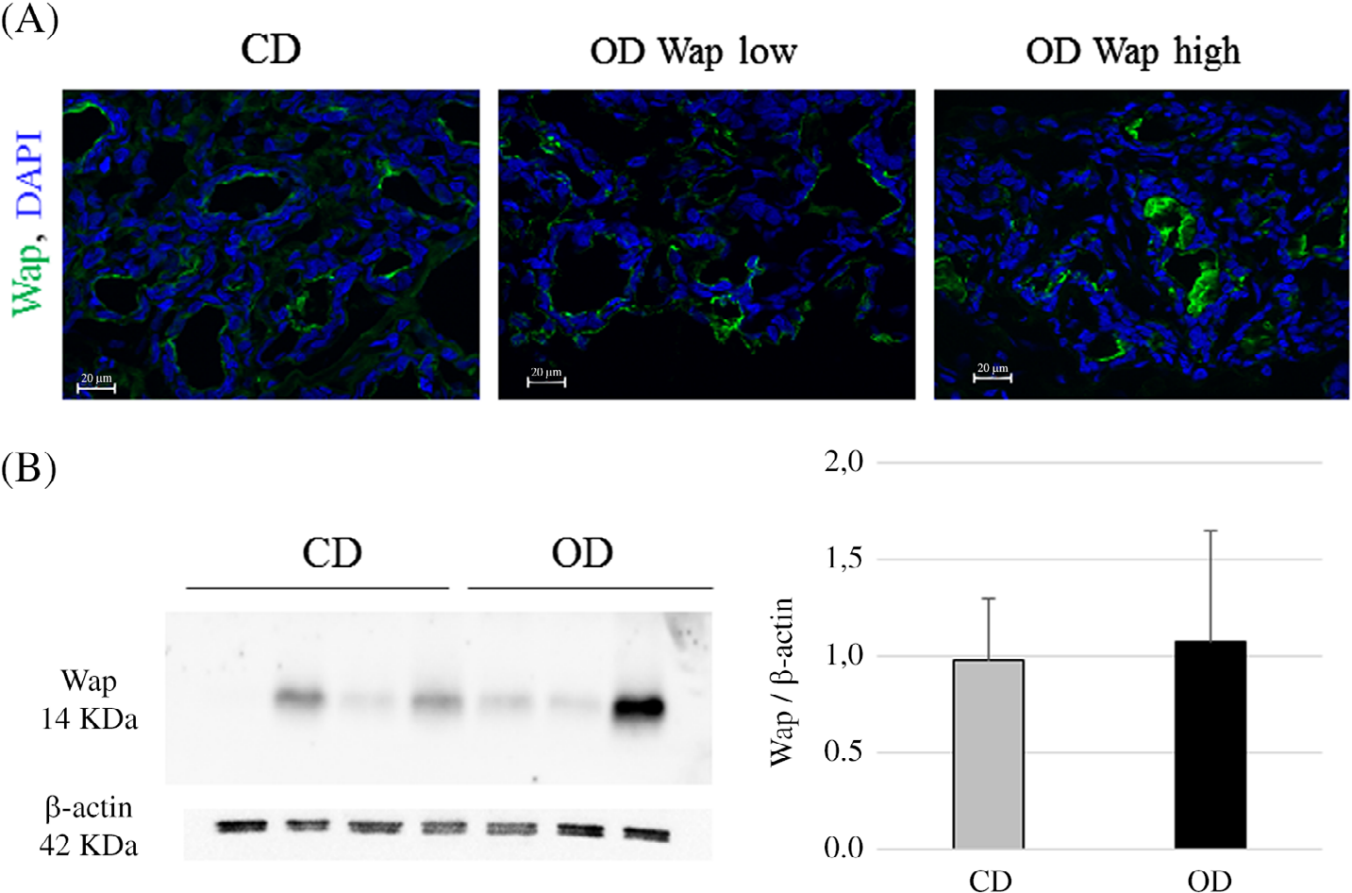
Immunolocalization and quantitative expression of Wap protein in rabbit mammary tissue on Day 8 of pregnancy. (A) Wap localization (green) was performed in CD and OD mammary tissue. DAPI was used to stain the nuclei (blue). Scale bar = 20 μm. (B) Western blot analysis of Wap and β‐actin proteins in CD and OD mammary gland and histogram quantification. Quantification was performed with all animals (CD: n = 9; OD: n = 7); one representative blot is shown. Data are expressed as means ± SEM

To further investigate MEC function, we analyzed the patterns of genes involved in milk protein synthesis using RT‐qPCR (Figure 5). The *kappa casein*, *Whey acidic protein* (*Wap*), and *alpha‐lactalbumine* (*Lalba*) milk protein encoding genes are specific markers for MEC differentiation. Although no differences in the expression of the *kappa casein* and *Lalba* genes were observed between the two groups, a significant increase in *Wap* expression occurred in the MEC of OD rabbits (Figure 5). Protein Wap study were performed by immunostaining and western blot analyses on CD and OD mammary glands. No difference was observed in protein level between the two groups (Figure 6). However, important variability between animals within the same group was noticed on both immunostaining analyses (Figure 6A) and blot quantification (Figure 6B).

Lipid metabolism in MEC was assessed from the expression of the *Stearoyl‐coA desaturase* (*Scd*) gene, which is involved in the fatty acids biosynthesis and has been shown to be more expressed in mammary epithelial tissue of high carbohydrate‐diet fed animals. We also analyzed the expression of *Fatty acid synthase N (FasN)*, which is found in cell types with a high lipid metabolism, and also in MEC.^31^ Among the genes tested, no significant modifications to their expression profiles were observed in the OD group compared to the CD group (Figure 5).

We also analyzed markers involved in the differentiation process affecting alveolar development during pregnancy, such as leptin or E74‐like factor 5 (Elf5). Leptin produced by MEC acts as an autocrine and paracrine factor to influence mammary gland development and differentiation,^32^ while Elf5 regulates alveolar differentiation and has been shown to be essential to the differentiation phase of alveolar development during pregnancy.^33^ The unbalanced diet restricted to the peri‐pubertal period did not affect *Elf5* expression (Figure 5).

**Figure 7:**
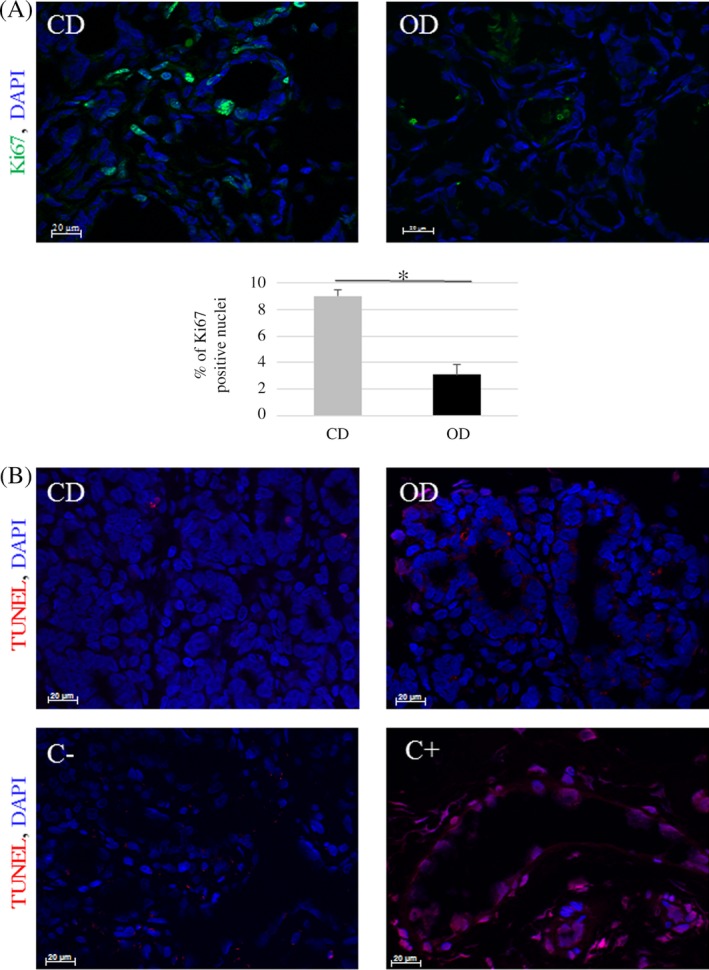
Proliferative and apoptotic status of mammary epithelial tissus on Day 8 of pregnancy. A, Ki67 immunostaining (green) was performed on mammary sections in CD and OD animals. Histogram summarizes the percentage of Ki67 positive nuclei in all animals (CD: n = 9; OD: n = 7) of both groups. Concerning tData are expressed as means ± SEM. Significant differences (at least *P* < .05) between the groups are indicated by asterisks (*). (B) TUNEL staining (red) was performed on mammary sections of CD and OD animals. Negative (C−) and positive (C+) controls are shown. DAPI was used to stain the nuclei (blue). Magnification ×400. Scale bar = 20 μm

We also assessed putative differences in cell proliferation between the two groups. The expression of proliferative markers such as the *Ki67* and *integrin‐β1* (*Itgb1*) genes, which play a key role in regulating the cell cycle progression of MEC, did not display any significant differences between CD and OD mammary tissue on Day 8 of pregnancy (Figure 5). Proliferative status of epithelial cells were estimated by Ki67 protein staining and positive nuclei counting.^34^ When the number of Ki67 positive nuclei was measured, a significant lower proliferation rate was observed in OD animals (Figure 7A). Moreover, TUNEL experiments were performed to estimate the apoptosis level. No difference was observed between CD and OD groups (Figure 7B).

**Figure 8:**
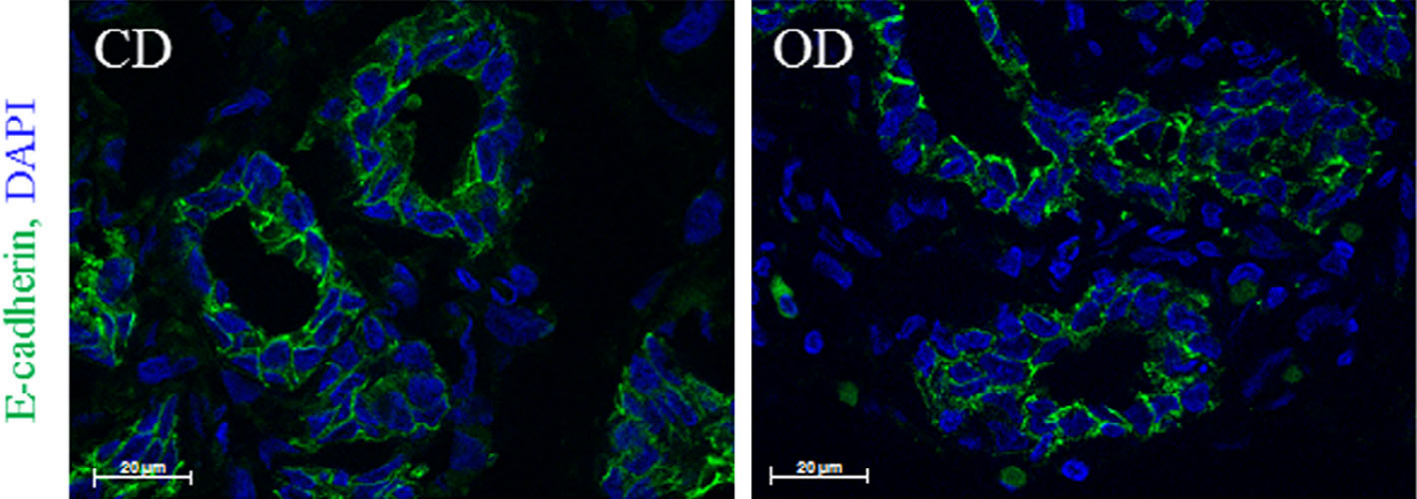
Immunolocalization of E‐cadherin in rabbit mammary tissue on Day 8 of pregnancy. E‐cadherin localization (green) was performed in CD and OD mammary tissue. DAPI was used to stain the nuclei (blue). Scale bar = 20 μm

Although histological analysis did not reveal any changes to the mammary epithelium, we investigated the integrity of MEC. One frequently studied molecular component of the adherent junctions is the adhesion receptor E‐cadherin (Cdh1), which is expressed in MEC during pregnancy.^35^, ^36^ At 8 days of pregnancy, Cdh1 expression was below the detection threshold in LCM‐captured MEC. However, E‐cadherin was detected by immunolabelling, in basolateral position of MEC in both CD and OD mammary tissue (Figure 8). No difference in Cdh1 localization has been observed between the two groups.

We therefore investigated the integrity of the MEC and their ability to produce milk components by analyzing markers such as Zona Occludens‐1 (ZO‐1) and Connexin 26 (Cx26). ZO‐1 is a specific component of adherent junctions, and Cx26 is a dominant gap junction protein found in MEC during pregnancy. *ZO‐1* expression in MEC was not modified by the peri‐pubertal diet (Figure 5). In contrast, *Cx26* expression was higher in the OD group than in the CD group (Figure 5). This result supports the hypothesis that nutritional alterations during the peri‐pubertal period alone may exert an effect on mammary epithelial function during early pregnancy.

## 3 DISCUSSION

The aim of this study was to better understand the long‐term consequences of nutrition during the period around puberty, with particular focus on mammary development. We therefore analyzed functional mammary epithelial tissue later in life, during early pregnancy. The high‐fat/high‐sugar diet used for our experiments had already been shown to induce mammary defects when administrated before puberty and then continued throughout mid‐pregnancy.^8^

At puberty, the mammary gland is a potent target for environment effects, particularly those related to nutrition, because the mammary epithelium has entered a stage of growth,^37^ leading to the establishment of structures that will differentiate to produce milk components.^38^ In early pregnancy, interstitial adipose tissue gradually disappears and proliferating epithelial cells fill in the interductal spaces and start to express genes that can be considered as differentiation markers, such as milk protein genes.^1^, ^39^ Changes to mammary development during pregnancy can impact the epithelial cell population that is responsible for the synthesis and secretion of milk components in lactation.^40^

During our experiments, OD females showed no difference in body weight compared to CD females, and this ongoing effect was associated with no difference in food intake between the two groups. These results are consistent with those observed previously in young ages when rabbits where fed with OD.^8^ Nor did OD exposure during the peri‐pubertal period affect either the metabolic profile or leptinemia in adulthood. These findings showed that exposure for 5 weeks to OD was not sufficient to induce obesity or metabolic syndrome in the rabbit. Similar results have been found in swine fed with an OD during the juvenile period; the gilts did not exhibit any signs of obesity, because they developed an adaptive response to the diet by reducing their food intake.^41^ By contrast, the feeding of rats with a high‐fat diet for 2 weeks around puberty induced significant adiposity and body weight gain.^42^ Similarly, in humans, an unbalanced dietary pattern during adolescence has been shown to cause greater adiposity and an increased metabolic risk.^43^

No differences were measured regarding the number of fetuses or fetal mortality in OD‐fed females compared to CD group, suggesting that OD exposure during the peri‐pubertal period did not affect reproductive performance, at least in early pregnancy. Although some studies have showed an impact of juvenile nutrition on ovarian development, as well as demonstrating the effect of nutrition on the onset of puberty, no studies have directly linked the influence of an unbalanced diet restricted to puberty with reproduction rates and mortality measured in early pregnancy.^25^, ^41^, ^44^

The effects of pre‐ and peri‐pubertal overfeeding on growth rate and mammary development have been investigated in heifers and it was found that an increased growth rate was due to high feeding levels before the onset of puberty, which thus led to reduced mammary growth and reduced milk yield potential.^45^ In contrast, feeding prepubertal heifers with a high‐fat diet increased the mammary fat pad and improved mammary development later in life.^27^ The ingestion of an OD during the peri‐pubertal period, as described here, led to a reduction in epithelial and connective tissues and an increase in adipose tissue. During the first half of pregnancy and under physiological conditions, adipose tissue levels diminish, while the number and size of alveoli rapidly increase.^46^ Using the same rabbit model, a previous study demonstrated the maintenance of mammary adipose tissue when OD was consumed from puberty to pregnancy.^8^, ^9^, ^14^ Taken together, these data support the evidence that a restricted window of susceptibility, such as puberty, may be sufficient to promote the development of mammary epithelial tissue. Moreover, other authors have produced evidence that overfeeding prepubertal heifers with a high‐energy diet reduced mammary epithelium mass at puberty and negatively affected mammary development by decreasing both MEC proliferation in areas of active ductal expansion and the mass of fat‐free mammary epithelial tissue.^47^, ^48^

To determine whether the reduction in alveolar epithelial tissue, observed in the present study, was due to either a decreased proliferation or an increased apoptosis of epithelial cells, measurements of Ki67 expression and TUNEL experiments have been used.^34^ Although *Ki67* is expressed in early pregnancy, we did not observe a significant difference in *Ki67* mRNA content between OD and CD‐fed rabbit mammary glands. However, the Ki67 reduced staining in the OD group, suggested that the lower representation of alveolar epithelium was due to a reduced proliferation of epithelial cells. Such discrepancy between transcript and protein expressions was already reported with Ki67 in breast cancer.^49^ We also investigated *Itgb1* expression because of its implication in mammary proliferation.^50^ In the OD group, epithelial Itgb1 expression did not differ from that seen in CD animals. Moreover, OD during puberty did not modify apoptotic status of the mammary epithelium on Day 8 of pregnancy.

The expression of enzymes involved in lipid metabolism in the mammary gland during the proliferation and differentiation steps was characterized, because alterations to lipid metabolism in MECs have been described in breast cancer.^51^ No differences were observed between the OD‐ and CD‐fed groups regarding the genes involved in lipid metabolism and lipogenesis. *FasN* expression was unchanged, suggesting that active fatty acid synthesis, which is required for energy utilization and membrane synthesis, was expressed equally in CD and OD mammary epithelia. The expression of *Scd*, which is involved in de novo lipid synthesis,^52^ was not impacted by exposure to OD around puberty. However, its expression has been shown to be modified by diet in adipose tissue.^53^ In an underfed sheep model, a reduction in *Scd* transcripts was observed, while no modifications to *Scd* expression were seen in the adverse overfed model.^54^ However, although feeding over 3 weeks was sufficient to increase the expression of *Scd* transcripts in the mammary gland of dairy goats fed a quality modified diet,^15^ an unbalanced diet during the peri‐pubertal period did not modify mammary lipid metabolism during early pregnancy in our model.

Markers for differentiation such as *κ casein*, *Wap*, *Lalba*, and *Elf5*, which plays an important role in mammary epithelial differentiation, are expressed in the mammary gland during pregnancy and lactation.^55^, ^56^ Our experiments revealed a significant increase in *Wap* expression, although significant variability was noted at the protein level. These results are in accordance with previous studies in the rabbit during pregnancy, showing that lactogenic hormones may function by increasing the rate of lactoprotein mRNA synthesis rather that initiating their transcription.^57^ Previous experiments in rodents had shown that dietary fatty acids prior to and during puberty altered the proliferative pathways in MEC and less frequently a few genes involved in the cell cycle pathway in adulthood.^58^ Our findings are of importance because they highlight the effect of a diet on the expression of genes that are essential either for epithelial development or for subsequent functions in pregnancy and later lactation. These effects were not observed with less unbalanced diets.^24^, ^59^ We also showed that OD during the peri‐pubertal period did not alter *Elf5* expression. However, the differentiation of alveolar tissue was enhanced by the diet consumed, as illustrated by increased *Wap* gene expression. Our results are consistent with those of Dewi et al, who reported an absence of diet‐induced effects on the gene expression of differentiation markers in a nonhuman primate model exposed to a soy diet around puberty.^59^

Cell to cell adhesion is required for the establishment and maintenance of the epithelial cell polarity that characterizes functional mammary epithelium during pregnancy and lactation.^60^ One of the principal contributors to epithelial integrity is the ZO‐1 adherent junction that mediates intercellular interactions.^61^ Studies have suggested that ZO‐1 may also promote the assembly and function of these junctions during epithelial morphogenesis.^62^ Recent work has shown that in a rodent model, the apoptotic event is preceded by a rapid loss of ZO‐1, and this loss of cell‐cell communication may initiate the involution and apoptosis of MEC.^63^ In our study, no modifications to ZO‐1 expression were observed in the MECs of OD‐fed animals compared to the controls, suggesting that the low level of epithelium in OD mammary tissue was not due to apoptosis but rather to less marked proliferation. A previous study showed that deletion of the *Cx26* gene in mammary epithelium before puberty dramatically compromised alveolar development during pregnancy and their function during lactation.^64^ During our experiments, we revealed a weak but significant increase of *Cx26* expression in epithelial cells at early pregnancy in OD‐fed rabbits. Up‐regulation of the *Cx26* gene has already been observed in cellular models following hormonal treatments, as well as during late pregnancy and lactation in different models.^65^, ^66^, ^67^ Our results suggest that the consumption of an OD during the peri‐pubertal period may, in early pregnancy, enhance the expression of genes that are usually increased in late pregnancy, thus leading to the hypothesis that an unbalanced diet during just the period of puberty might promote enhanced signs of MEC development and functions.

Taken together, our results demonstrate that exposure to an OD limited to the peri‐pubertal period enhances the differentiation of MEC, as illustrated by the expression of specific markers. It is particularly important to elucidate these critical nutritional periods when considering their long‐term consequences on the health and development of the mammary gland and its functions, and the promotion of mammary cancer development in adulthood. Some studies have correlated a high‐fat prepubertal diet with a risk of breast cancer and found opposite effects depending on the animal model and mainly on the fat content of the diet.^20^ In mouse models, a peri‐pubertal high‐fat diet is sufficient to irreversibly promote mammary carcinogen‐induced tumorigenesis in adulthood.^19^ Nevertheless, our work has highlighted the potentially important role played by unbalanced nutrition during critical early‐life windows in terms of regulating long‐term differentiation and mammary function.

## 4 EXPERIMENTAL PROCEDURES

### 4.1 Animals, experimental design, and sampling

This study was carried out in compliance with the French regulations on animal experimentation and with the authorization of the French Ministry of Agriculture. All protocols were approved by an Ethics Committee registered within the French Comité National de Réflexion Ethique sur l'Expérimentation Animale. The protocol referred to above was approved (visa APAFIS#5600‐2 016051218112190 v4) by the Comité d'éthique appliqué à l'Expérimentation Animale (COMETHEA Ethics Committee).

Sixteen female rabbits (New Zealand White, 1077‐INRA) were housed individually in an indoor facility under controlled conditions of temperature (18°C) and light (usually an 8/16 hours light/darkness cycle except for an inverted cycle during the week before mating). Before puberty, at the age of 8 weeks, the rabbits were divided randomly into two groups and fed ad libitum with the previously described CD or OD^8^ for 5 weeks. From the age of 13 weeks, both groups received the same CD. Growth was determined by weighing the rabbits once a week from weaning to the age of 20.5 weeks, and food intake was also monitored on the same weekly basis. Blood samples were collected at the age of 20 weeks in tubes containing EDTA to determine levels of triglyceride (Triglyceride PAP RTU kit; Biomerieux), cholesterol (Cholesterol RTU kit; Biomerieux), glucose (Glucose RTU kit; Biomerieux), and leptin (Cloud Clone Corp.).

At 21 weeks of age, the females were mated with CD‐fed males and then sacrificed on Day 8 of pregnancy. The left lower mammary gland from each animal was excised and dissected and mammary samples were processed and stored for further analyzes. The placentas were dissected and the fetuses numbered.

### 4.2 Histological analysis

In each animal on Day 8 of pregnancy, the left lower mammary gland was excised and divided into several samples. Mammary samples were dissected to remove muscle. For histology, they were fixed for 24 hours in RCl2 buffer (Alphelys, France) before embedding in paraffin. Five‐micrometer sections, at least 100 μm apart, were mounted on slides. The slides were stained with hematoxylin and eosin (H&E; Sigma‐Aldrich) and then digitized under bright light using a Hamamatsu NanoZoomer (Hamamatsu Photonics). Eight sections per rabbit were processed and areas occupied by mammary epithelial tissue (clusters of alveolar structures), adipose tissue, mammary duct lumens, or connective tissue were measured using CaseViewer software (3D Histech) and divided by the whole section area to generate the proportion of each tissue. Results are expressed as means ± SEM.

### 4.3 Immunostaining and TUNEL assay

For immunohistochemical detection of Ki67, heat‐induced retrieval was performed by micowaving sections in 10 mM sodium citrate, pH 6.0 for 10 minutes. After blocking in 0.05% heat‐inactivated fetal calf serum, sections were incubated overnight at 4°C with primary antibody (Mouse monoclonal anti‐Ki67, 1:200, Dako), followed by incubation for 1 hour at room temperature with secondary antibody (donkey anti‐mouse FITC‐conjugated 1:300), then counterstained with Vectaschield‐DAPI medium (Vectorlabs).

TUNEL staining was carried out on 5 μm paraffin sections using the in situ Cell death Detection kit (Roche Aplied Science) according to manufacturer's recommendations.

The Wap and Cdh1 protein were localized by immunohistochemical analyses using the following dilutions of primary antibodies: guinea pig anti‐rabbit Wap (1:500, [68]) and mouse monoclonal anti‐E‐cadherin (1:200, Becton Dickinson Laboratories). Briefly, 5‐mm frozen sections were treated in 50 mM ammonium chloride for 30 minutes followed by permeabilization in 2% BSA, 0.05% saponin, and 0.05% sodium azide in PBS 1_ for 1 hour. Primary antibodies were diluted in the same buffer and then added to the tissue sections for 1 hour at room temperature. Antibody binding was visualized with fluorescence‐labeled secondary antibodies (anti‐guinea pig FITC‐conjugated, 1:300, and anti‐mouse FITC conjugated, 1:300, Jackson Immunoresearch), applied to the sections in PBS 1_ for 45 minutes. Slides were mounted in Vectashield‐DAPI medium (vector Laboratories).

The immunofluorescence was viewed under a Zeiss Apotome microscope, at magnification ×400, and the quantification of Ki67 and TUNEL labelled nuclei was performed using the ImageJ software using five pictures per animal, captured randomly in the epithelial area of the sections. Results are expressed as means ± SEM.

### 4.4 Western blot analysis

Protein extracts from each rabbit mammary gland were prepared in 50 mM Tris‐HCl (pH 8), 137 mM NaCl, 2.7 mM KCl, 1% NP40, and 10% glycerol, containing protease and phosphatase inhibitors (Complete Mini and PhoSTOP, Roche, Meylan, France). Their protein concentration was determined using the BCA Protein Assay kit (Thermo Scientific, France). Proteins (5 μg) were separated on 10% SDS‐polyacrylamide gel and transferred onto nitrocellulose filters (Bio‐rad, France). The membranes were saturated for 1 hour in blocking solution (TBS‐T:50 mM Tris pH 7.4, 200 mM NaCl, 0.1% Tween 20, 5% defatted milk) and incubated overnight with the primary antibodies at 4°C. After incubation, the membranes were washed with TBS‐T and incubated at room temperature with horseradish peroxidase‐linked secondary antibody for 45 minutes. Immune complexes were detected using the ECL kit for autoradiography (GE Healthcare, France). The following specific antibodies were used: guinea pig anti‐rabbit Wap (1:5000) antibodies,^68^ and anti‐β‐actin mouse antibody (1:10 000, Clone AC‐15, Sigma‐Aldrich, France) as loading control. The secondary antibodies used for immunoblotting were: anti‐guinea pig (1:5000) and anti‐mouse (1:10 000) horseradish peroxidase (HRP)‐conjugated (Sigma‐Aldrich, France). Quantification was assayed using ImageJ software and results are expressed as means ± SEM.

### 4.5 Laser capture microdissection

In order to study the gene expression profile of mammary epithelial tissue, the technical isolation of epithelium using laser microdissection of the rabbit pregnant mammary gland was adapted.^28^ Briefly, mammary samples were collected aseptically from the CD‐fed (N = 9) and the OD‐fed dams (N = 7), 10 minutes after slaughtering. A 5 mm^3^ piece of alveolar parenchyma, without connective tissue and muscle, was washed in cold PBS solution, placed on ice and embedded in OCT (TissueTek) in a 1 cm^3^ cryomold (Bayer) and then immersed immediately in liquid nitrogen vapor for freezing. The samples were stored at −80°C until further processing. Eight micrometer tissue cryosections were cut and mounted on cold slides and stored in 70% ethanol at −20°C. The slides were stained with cresyl violet and dehydrated, and then LCM was carried out using the Veritas Microdissection system and software (Arcturus, Life Technologies).

### 4.6 RNA extraction and RT‐qPCR

Total RNA from captured mammary epithelial tissue was isolated from each sample using the RNA NOW kit (Ozyme) according to the manufacturer's protocol. The integrity of ribonucleic acid was assessed using an Agilent Bioanalyzer. Samples with an RNA Integrity Number (RIN) higher than 7 were subsequently studied.

For gene assays by quantitative PCR (qPCR), reverse transcription (RT) was performed on 200 ng of total RNA using the SuperScript VILO cDNA Synthesis kit according to the manufacturer's instructions (Invitrogen) and under the following conditions: 42°C for 60 minutes and 85°C for 5 minutes.

qPCR runs were achieved using Applied Biosystems SYBR Green PCR Mastermix (Thermo Scientific) according to the manufacturer's instructions, on a QuantStudio system (Thermo Scientific). After the optimization of qPCR systems (efficiency ranging from −3.25 to −3.45), amplification reactions were run in triplicate under the following conditions: 95°C for 15 minutes, 45 cycles of 95°C for 15 seconds and 60°C for 1 minute. The threshold cycles obtained for each gene were normalized with the values of the *TATA Binding Protein (Tbp)* gene and the results were expressed as fold changes of the threshold cycle (Ct) values relative to the control using the 2‐ΔΔCt method.^69^ The primers used for each gene amplified are presented in Table S1.

### 4.7 Statistical analyses

Data are expressed as means ± SEM. The effects of diet during the peri‐pubertal period on histological features (stereology) and mRNA (RT‐qPCR) expression in the mammary gland were assessed by the nonparametric Mann Whitney *U* test using StatE1 software (AD Science). *P*‐values of <.05 were considered to be statistically significant. When necessary, statistical analyses were supplemented by an ANOVA approach, using the “aovp” function of the “ImPerm” package (http://cran.rproject. org/package = ImPerm). This approach is analogous to a conventional ANOVA except that *P*‐values are obtained by pairwise permutations of the data rather than being derived from *F*‐tests. The use of permutation ANOVA is particularly suitable for data sets with relatively few replicates and ensures robust tests with respect to violated classical ANOVA assumptions (homoscedasticity and normality).

## Supporting information


**Table S1** Primer sequences used for qPCR experiments
