# Supplementary material for: Puberty is a critical window for the impact of diet on mammary gland development in the rabbit
Source: Dev Dyn. 2019 Aug 2;248(10):948–60. doi: 10.1002/dvdy.91 (PMC6790954; doi:10.1002/dvdy.91)
Supplement: Supplementary file 1 — Table S1 Primer sequences used for qPCR experiments [file DVDY-248-948-s001.docx]

**Supplementary Table 1.** Primer sequences used for qPCR experiments

| Genes | Primer | Sequence 5’🡪3’ |
| --- | --- | --- |
| *Kappa casein* | Forward | GGAACAGACAACGTGCCGTG |
|  | Reverse | CGAACCCAGCTACTACCTGC |
| *Whey acidic protein (Wap)* | Forward | T GCGCTATCTGGAACCCATC |
|  | Reverse | GAGAGTTGGGCCTGAGTTCC |
| *Alpha-lactalbumin (Lalba)* | Forward | AT CAGCGATAAGCTGTGGTGT |
|  | Reverse | ATTG ACCACTGGTTGGCACAT |
| *Fatty acid synthase N (FasN)* | Forward | ACCTCGTGAAGGCTGTGACTCA |
|  | Reverse | TGAGTCGAGGCCAAGGTCTGAA |
| *Stearoyl-coA desaturase (Scd)* | Forward | TTATTCCGTTATGCCCTTGG |
|  | Reverse | TTGTCATAAGGGCGGTATCC |
| *Keratin 8 (Krt8)* | Forward | AACTACGGCCTGAGTTTCCAG |
|  | Reverse | GCCTTGGAGCGGGTAAAGGA |
| *Keratin 14 (Krt14)* | Forward | CTGGGTAGCGCCTATGGT |
|  | Reverse | CTGCATGGTCACCTTCTCACT |
| *E74-like factor 5 (Elf5)* | Forward | GTGGACCGATCTGTTCAGCA |
|  | Reverse | GGTCCAATAGGAGTCGCAGG |
| *E-cadherin (Cdh1)* | Forward | CAATTCCAGGGGCTCACTCC |
|  | Reverse | TGGCCATGAAAGGAAGTCTCC |
| *Ki67* | Forward | CCATCCTGGACATCGTGACA |
|  | Reverse | GGGAGATTCTTCTTTCCGGCT |
| *Integrin beta-1 (Itgb1)* | Forward | GTCTGTGGGTCGCTGATCG |
|  | Reverse | ACGAGGTGGGCAATAGAAGGG |
| *Connexin 26 (Cx26)* | Forward | TGAGGCCGTCTTCATGTACG |
|  | Reverse | CAGGCATTGCACTTCACCAG |
| *Zona Occludens 1 (ZO-1)* | Forward | GTCTGCCATTACACGGTCCT |
|  | Reverse | GGTCTCTGCTGGCTTGTTTC |
| *TATA Binding protein (Tbp)* | Forward | TGACCCCCATGACCCCTATT |
|  | Reverse | CAGCAAACCGCTTGGGATTA |
